# Selective Recruitment of Presynaptic and Postsynaptic Forms of mGluR-LTD

**DOI:** 10.3389/fnsyn.2022.857675

**Published:** 2022-05-09

**Authors:** Thomas M. Sanderson, Liam T. Ralph, Mascia Amici, Ai Na Ng, Bong-Kiun Kaang, Min Zhuo, Sang Jeong Kim, John Georgiou, Graham L. Collingridge

**Affiliations:** ^1^Lunenfeld-Tanenbaum Research Institute, Mount Sinai Hospital, Sinai Health System, Toronto, ON, Canada; ^2^Department of Brain and Cognitive Sciences, College of Natural Sciences, Seoul National University, Seoul, South Korea; ^3^Neuroscience Research Institute, Seoul National University College of Medicine, Seoul, South Korea; ^4^School of Physiology, Pharmacology and Neuroscience, University of Bristol, Bristol, United Kingdom; ^5^Department of Physiology, Temerty Faculty of Medicine, University of Toronto, Toronto, ON, Canada; ^6^Department of Biological Sciences, College of Natural Sciences, Seoul National University, Seoul, South Korea; ^7^Tanz Centre for Research in Neurodegenerative Diseases, University of Toronto, Toronto, ON, Canada

**Keywords:** long-term depression (LTD), metabotropic glutamate (mGlu) receptor, DHPG, hippocampus, CA1

## Abstract

In area CA1 of the hippocampus, long-term depression (LTD) can be induced by activating group I metabotropic glutamate receptors (mGluRs), with the selective agonist DHPG. There is evidence that mGluR-LTD can be expressed by either a decrease in the probability of neurotransmitter release [P(r)] or by a change in postsynaptic AMPA receptor number. However, what determines the locus of expression is unknown. We investigated the expression mechanisms of mGluR-LTD using either a low (30 μM) or a high (100 μM) concentration of (RS)-DHPG. We found that 30 μM DHPG generated presynaptic LTD that required the co-activation of NMDA receptors, whereas 100 μM DHPG resulted in postsynaptic LTD that was independent of the activation of NMDA receptors. We found that both forms of LTD occur at the same synapses and that these may constitute the population with the lowest basal P(r). Our results reveal an unexpected complexity to mGluR-mediated synaptic plasticity in the hippocampus.

## Introduction

Group I metabotropic glutamate receptor-dependent long-term depression (mGluR-LTD) is a widely studied form of synaptic plasticity that is required for certain learning and memory tasks (Collingridge et al., 2010; Luscher and Huber, 2010; Goh and Manahan-Vaughan, 2013; Di Prisco et al., 2014; Eales et al., 2014). This plasticity is necessary for healthy brain function as it is dysregulated in several models of neurodevelopmental disorder (Bear et al., 2004; Hsieh et al., 2006; Mameli et al., 2007; Auerbach et al., 2011; Chevere-Torres et al., 2012).

Despite the importance of mGluR-LTD in both health and disease, the mechanisms that underlie its expression are not completely understood. A widely used method of inducing this form of synaptic plasticity, application of the group I mGluR agonist (R,S)-3,5-Dihydroxyphenylglycine (DHPG), results in either the synaptic removal of AMPA receptors (AMPARs) (Snyder et al., 2001; Xiao et al., 2001; Nosyreva and Huber, 2005; Moult et al., 2006; Sanderson et al., 2011, 2018) or a reduction in the probability of neurotransmitter release [P(r)] (Fitzjohn et al., 2001a,b; Watabe et al., 2002; Rammes et al., 2003; Tan et al., 2003; Moult et al., 2006; Sanderson et al., 2011). How these two types of DHPG-induced LTD (DHPG-LTD) are related is unknown.

Since its first description (Palmer et al., 1997) it has been known that there are at least two forms of DHPG-LTD that can be distinguished on the basis of their sensitivity to NMDA receptor (NMDAR) antagonists. A low concentration of DHPG induces a form of LTD that requires co-activation of NMDARs whereas a high concentration of DHPG induces an NMDAR-independent form of LTD. We hypothesized that these two distinct forms of LTD have different loci of expression. We tested this idea by recording evoked and miniature EPSCs in rat organotypic slices and fEPSPs in acutely prepared rat hippocampal slices. We found that 30 μM DHPG induced an NMDAR-dependent presynaptic form of LTD. In contrast, we found that 100 μM DHPG induced an NMDAR-independent postsynaptic form of LTD and was associated with the shrinkage of spines. Furthermore, these two mechanistically distinct forms of LTD impact the same set of synapses, which we propose are those with the lowest P(r).

## Materials and Methods

### Preparation of Organotypic Slice Culture

Organotypic hippocampal slices were prepared as described previously (Sanderson et al., 2018). Briefly, male P7 SD rats were sacrificed by cervical dislocation in accordance with the UK animals (Scientific procedures) act 1986 and the South Korean Animal Protection act, 1991. Hippocampi were removed and 350 μm thick slices were prepared using a Leica VT 1200S vibratome (Leica microsystems, Germany). Slicing was performed in cutting solution containing (in mM) sucrose (238); KCl (2.5); D-glucose (11); NaHCO_3_ (26); NaH_2_PO_4_ (1); CaCl_2_ (1) and MgSO_4_ (5). Slices were then washed in filtered culture media containing 78.8% minimal essential media (MEM) with Hank’s salts; 20% horse serum; HEPES (30); D-glucose (16); NaHCO_3_ (5); CaCl_2_ (1); MgSO_4_ (2); L-ascorbic acid (0.68) and insulin (1 μg/ml). Slices were then placed on sterile 30 mm Millicell cell culture inserts (0.4 μm pore size, Millipore, Ireland) resting on culture media and maintained at 35°C in 5% CO_2_. All equipment was sterilized before use. Media was changed every 2 days. Slices were used at 2–3 weeks *in vitro*.

### Patch Clamp Electrophysiology

Patch clamp electrophysiological recordings were performed on organotypic hippocampal slices. In brief, slices were transferred to a recording chamber and continuously perfused with artificial cerebral spinal fluid (ACSF) containing (in mM): NaCl (119); KCl (2.5); D-glucose (11); NaHCO_3_ (26); NaH_2_PO_4_ (1); CaCl_2_ (4); MgCl_2_ (4); picrotoxin (0.05) and 2-chloroadenosine (0.01) at 2 mL/min, heated using an inline heater to 33°C. 2-chloroadenosine was included to reduce excitability. Where mEPSCs were recorded, tetrodotoxin citrate (0.5 mM) was also included in the ACSF. Patch electrodes (∼3–5 MΩ) were used that contained whole-cell solution comprised of (in mM): cesium-methane sulphonate (130); HEPES (10); EGTA (0.5); NaCl (8); Mg-ATP (4); Na_2_-GTP (0.3); QX314 (6); ∼290 mOsm/L; pH adjusted to 7.2 using CsOH. The initial offset potential was corrected before recording and cells were held at –70 mV. In paired pulse experiments the inter stimulus interval was 50 ms. Recordings were sampled at 20 kHz using WinLTP (Anderson and Collingridge, 2007; Anderson et al., 2012), filtered at 2 kHz and series resistance was measured at 30 s intervals throughout recordings. Recordings in which the series resistance varied by <20% were accepted for analysis. Evoked EPSCs were analyzed offline using WinLTP. AMPAR-mediated synaptic transmission peak responses at -70 mV were measured. Miniature excitatory postsynaptic currents (mEPSCs) were analyzed using Minianalysis (Synaptosoft Inc., Decatur, GA, United States).

### Animals and Slice Preparation for *ex vivo* Electrophysiology

Young postnatal day 14–21 wild-type rats (Sprague-Dawley) were anaesthetized with isoflurane and sacrificed by decapitation in accordance with the Canadian Council on Animal Care (CCAC) guidelines and AUPs approved by UHN and TCP Animal Care Committees. Brains were rapidly extracted and recovered for 3–5 min in chilled (0–4 °C) ACSF saturated with 95% O_2_ and 5% CO_2_. Dissection ACSF contained (in mM): 124 NaCl, 10 D-Glucose, 24 NaHCO_3_, 3 KCl, 1.25 NaH_2_PO_4_⋅H_2_O, 1 MgSO_4_, and 2 CaCl_2_. Hippocampi were extracted from each brain hemisphere and the ventral third was removed. The remaining tissue was transversely sliced 400 μm thick using a Leica VT1200 vibratome (1.2 mm/min speed, 2.0 mm amplitude). Slices recovered at room temperature for 1.5–2 h before the start of each experiment.

### *Ex vivo* Electrophysiological Recordings

For every *ex vivo* electrophysiological recording, slices were perfused with ACSF (which contained the same composition as the dissection ACSF but with the following modifications: 2 mM MgSO_4_ and 50 μM picrotoxin) delivered at 28 °C and 2.5 mL/min. The 30 μM DHPG and the 100 μM DHPG experiments were paired such that one slice per animal was used for each condition; therefore all N values represent the animal number per condition. Field-excitatory postsynaptic potentials (fEPSPs) were obtained by stimulating CA3 Schaffer collaterals to CA1 pyramidal neuron synapses in the stratum radiatum of dorsal-intermediate hippocampus slices. A bipolar matrix stimulation electrode (FHC, ME, United States) was placed 300 ± 25 μm away from a borosilicate glass recording electrode filled with recording ACSF (with a resistance in the range of 1.5–2.0 MΩ). Stimulus current pulses (duration of 0.1 ms) were generated using a stimulus isolating unit (STG 4002; MCS, Multichannel systems, Germany). Field EPSPs were evoked by stimulating once every 30 s and are presented as an average of four consecutive responses. Baseline stimulation intensity was set to 1.5 times the threshold for evoking fEPSPs and 0.1% DMSO or 10 μM L-689,560 were present for the entire baseline of 30 or 100 μM DHPG experiments, respectively. To induce mGluR-LTD, either 30 or 100 μM DHPG was applied for 10 min in the presence of 0.1% DMSO or 10 μM L-689,560. Paired-pulses 50 ms apart were delivered in triplicate and measured before baseline application of 0.1% DMSO or 10 μM L-689,560 and at 30 and 60 min after the end of 10 min of DHPG application. Recorded signals were sampled at 20 kHz, (Multiclamp 700B, Molecular Devices, United States), digitized at 2 kHz (National Instruments, United States), and acquired using WinLTP software (Anderson and Collingridge, 2007; Anderson et al., 2012). Analysis of fEPSPs were performed offline using Clampfit 10.6 (Molecular Devices, United States). The fEPSP slope was measured as 20–60% of the maximum response.

### Biolistic Transfection

Plasmid DNA was amplified using QIAGEN Plasmid Maxi Kits (QIAGEN, Manchester, United Kingdom). Gene gun bullets were prepared as described previously (Sanderson et al., 2018). In brief, spermidine (0.05 M), plasmid DNA and calcium chloride (1 M) were added to 1 μm diameter gold micro carriers (in that order) and incubated at room temperature for 10 min with intermittent agitation. 1–10 μg of DNA was added per mg of gold. Gold bullets were then washed with pure ethanol three times and suspended in polyvinylpyrrolidone (PVP) solution in ethanol (75 μg/mL). This solution was transferred to Tefzel tubing, the gold bullets were allowed to sediment at the bottom of the tube and the remaining solution was removed. Rotation of the tube caused its interior surfaces to be coated with gold bullets and a flow of nitrogen through the tube dried the bullets in place. The gold bullet coated tubing was segmented into 1 cm pieces, such that 0.2–0.5 mg of DNA coated gold particles were in each segment, and the gold bullets were fired at organotypic slices using a Helios gene gun (Biorad, CA, United States) with 140 psi helium. Organotypic slices were transfected after 1 week in culture, and were used in experiments at ∼2 weeks in culture.

### EGFP Imaging

CA1 pyramidal cells were transfected as described above and maintained in the same conditions as outlined for patch clamp experiments. We previously found that synaptic stimulation is necessary to observe DHPG-LTD^post^ (Sanderson et al., 2018). Accordingly, we provided axonal stimulation at 0.03 Hz at an intensity that induced synaptic currents, as observed using whole-cell recording (not shown). Images of living neurons were acquired using an Olympus FV1200MPE microscope (Olympus, Southend-on-sea, United Kingdom) in combination with a Mai Tai DeepSee multiphoton laser (Spectra Physics, Mountain View, CA, United States). An excitation wavelength of 900 nm was used. The minimum light intensity was used that resulted in adequate signal to noise ratio. The range of laser intensities used were between 20 and 70 mW, measured at the back aperture of the objective lens using a Spectra physics Model 407A power meter (Spectra Physics, Mountain View, CA, United States). Also, the total number of images was kept to the minimum so that bleaching was negligible. Image stacks consisting of 1 μm axial z-steps were constructed.

For analysis of EGFP fluorescence, average z projected images were constructed from the image stacks and regions of interest (ROIs) were placed over fluorescent puncta. Small movements of dendrites or spines were compensated for by replacing the ROIs manually at each time point. Integrated pixel density was measured to assess morphology as previously described (Woods et al., 2011; Stein et al., 2021).

### Modeling

We produced binary outputs (e.g., either 0 or 1) to represent successful or unsuccessful release events from 58 synapses, the likelihood of which depended on their P(r) based on FM 4–64 staining observed in Sanderson et al., 2018. We produced random number weighted probabilities using the MATCH and RAND functions in excel, and directed these using the INDEX function to the values 0 and 1. As described in https://exceljet.net/formula/random-number-weighted-probability, the formula was:


=INDEX(outputvalues,MATCH(RAND(),cumulative_probability))

The “output values” referred to cells that contained either 0 or 1 and “cumulative probability” referred to cells that contained the P(r) for each synapse expressed as a cumulative probability. For each synapse in the data set the outcome based on this formula was summed to produce a simulated EPSC. This procedure was repeated 10 times to produce mean and SEM values.

To produce a simulated PPF, the initial P(r) values were multiplied by a facilitation factor determined using a model that predicts the P(r) of the second pulse in a PPF experiment (Murthy et al., 1997). The formula used was:


$$f\,\left(p\right)=\frac{1-\left(1-p\right){}^{{}^{\frac{1}{\bar{p}}}}}{p}$$

Where *f* refers to the facilitation and *p* refers to the initial P(r). These facilitated P(r) values were then used to produce simulated EPSC values that modeled the synaptic responses generated by the second pulse in a PPF experiment. The simulated PPF values were determined by dividing this second simulated EPSC by the simulated EPSC produced using the initial P(r) values.

### Compounds

(R,S)-3,5-DHPG (HB0026), and picrotoxin (HB0506), were purchased from HelloBio Inc., United States). L-689,560 (0742) was purchased from Tocris Bioscience (Bio-Techne Canada). All compounds were prepared as stock solutions, aliquoted for single experiment use, stored frozen at −30°C and added to perfusing ACSF within 10 min of experiment initiation.

### Statistics

Data is presented as mean ± standard error of the mean (SEM). Statistical significance between 2 conditions was determined using paired or unpaired t-tests as appropriate, between 3 or more conditions was determined using a one-way ANOVA followed by post hoc Bonferroni correction. Where data was plotted as cumulative probability plots Kolmogorov-Smirnov tests were used. Significance was set at p < 0.05 and is indicated in the figures (^∗^p < 0.05, ^∗∗^p < 0.01, ^∗∗∗^p < 0.001).

## Results

### DHPG-LTD of Evoked Synaptic Transmission

**FIGURE 1:**
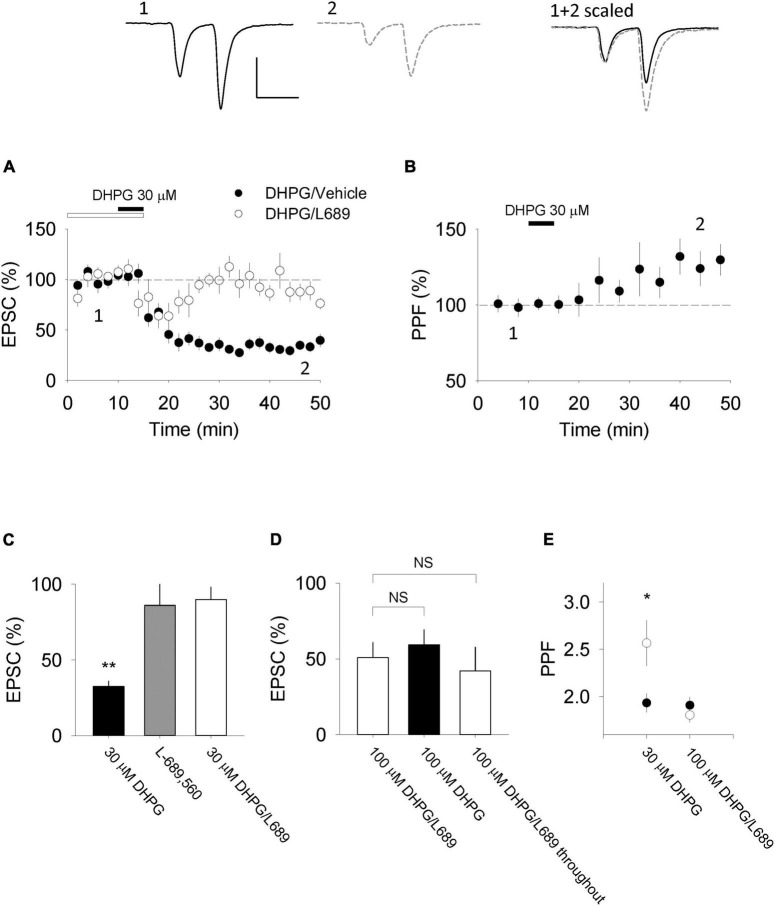
DHPG-LTD of evoked synaptic transmission in organotypic slices. **(A)** 30 μM RS-DHPG applied for 5 min results in LTD when applied alone but not when 10 μM L-689,560 is co-applied during induction (e.g., L-689,560 was washed out at the same time as DHPG). **(B)** DHPG-LTD induced by 30 μM DHPG is accompanied by an increase in PPF recorded at an inter stimulus interval of 50 ms. Upper traces show examples for experiments in which 30 μM RS-DHPG was applied alone [from panels **(A,B)**]. Scale bar is 100 pA by 50 ms. **(C)** Summary of data shown in panel A, together with a comparison with the effect of L-689,560 on evoked EPSCs when applied alone. Statistical significance determined using a paired *t*-test. **indicates *p* < 0.01. **(D)** Comparison of LTD induced by 100 μM DHPG applied in the presence of 10 μM L-689,560 where L-689,560 is washed out immediately after DHPG application (previously published in Sanderson et al., 2018), 100 μM DHPG applied alone and LTD induced by 100 μM DHPG applied in the presence of 10 μM L-689,560 where L-689,560 is not washed out. **(E)** Summary of PPF data shown in panel B together with a comparison of PPF changes induced by 100 μM DHPG applied in the presence of 10 μM L-689,560 where L-689,560 is washed out immediately after DHPG application (the 100 μM data was previously published in Sanderson et al., 2018). Statistical significance determined using a paired *t*-test. *indicates *p* < 0.05.

In organotypic slices we found that 30 μM DHPG induced a substantial LTD (to 35 ± 4% of baseline; *n* = 10, *p* < 0.01; Figures 1A,C), measured 30 min after the start of DHPG washout. This LTD was accompanied by an increase in paired-pulse facilitation (PPF) to 130 ± 10% of baseline (*p* < 0.05; Figures 1B,E) indicating that the LTD is most likely expressed *via* a decrease in P(r). We therefore refer to this form of LTD as DHPG-LTD^pre^. To examine the requirement for NMDAR activation, we tested a glycine site antagonist. L-689,560 (10 μM) alone did not significantly affect synaptic transmission evoked by baseline stimulation (86 ± 14%, *n* = 5, *p* > 0.05; Figure 1C but blocked the effects of DHPG, (88 ± 8% of baseline; *n* = 6; *p* > 0.05; Figures 1A,C). However, in interleaved experiments, increasing the DHPG concentration (from 30 to 100 μM) enabled a substantial LTD to be induced in the presence of L-689,560 (47 ± 10%; Figure 1D; *n* = 6, *p* < 0.05; see Sanderson et al., 2018). A similar level of LTD was also observed when L-689,560 was applied throughout (38 ± 13%, *n* = 3, *p* > 0.05; Figure 1D). In contrast to DHPG-LTD^pre^ this form of LTD was not accompanied by a change in PPF (99 ± 11%; *p* > 0.05; Figure 1E; see Sanderson et al., 2018) indicating that the LTD is not expressed *via* a change in P(r). This leaves a reduction in synaptic AMPARs as most likely expression mechanism, which we therefore refer to as DHPG-LTD^post^.

In a subset of DHPG-LTD^post^ experiments, we applied 100 μM DHPG alone in the absence of L-689,560. Surprisingly, the level of LTD (51 ± 8%, *n* = 3; Figure 1D) was not greater than that observed in the presence of L-689,560 (*p* > 0.05), indicating that DHPG-LTD^pre^ and DHPG-LTD^post^ are not additive.

**FIGURE 2:**
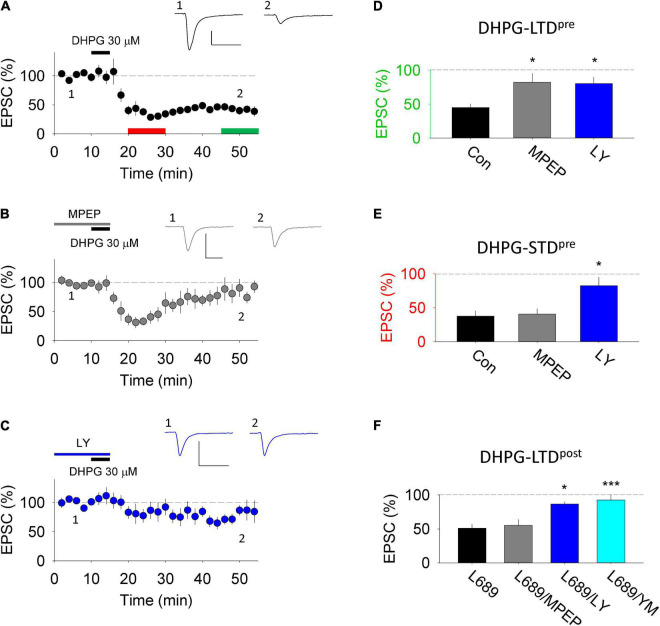
DHPG-LTD pharmacology in organotypic slices. **(A)** 30 μM RS-DHPG applied for 5 min (DHPG-LTD^pre^ protocol) results in LTD when applied alone. Scale, 100 pA by 50 ms. **(B)** 30 μM RS-DHPG applied for 5 min in MPEP results in reduced LTD without affecting the acute STD. Scale, 100 pA by 50 ms. **(C)** 30 μM RS-DHPG applied for 5 min in LY-367385 results in reduced LTD and STD. Scale, 100 pA by 50 ms. **(D)** Summary LTD data for panels **(A–C)**. Statistical significance determined using one-way ANOVA, * indicates *p* < 0.05. **(E)** Summary STD data for panels **(A–C)**. Statistical significance determined using one-way ANOVA, * indicates *p* < 0.05. **(F)** Summary data of LTD induced by 100 μM RS-DHPG applied in L-689,560 (DHPG-LTD^post^ protocol), together with the effect of applying MPEP, LY-367385 and YM298198 on this protocol. Previously published in Sanderson et al., 2018. Statistical significance determined using one-way ANOVA, * indicates *p* < 0.05, *** indicates *p* < 0.001).

DHPG activates the two group I mGluR subtypes, mGluR1 and mGluR5, to a similar extent (Conn and Pin, 1997). To determine the subtype(s) involved in DHPG-LTD^pre^ and DHPG-LTD^post^ we compared the effects of the mGluR1 antagonist, LY367385 (LY; 100 μM) and the mGluR5 antagonist, 2-Methyl-6-(phenylethynyl)pyridine (MPEP; 10 μM). DHPG-LTD^pre^, quantified 30 min after the start of washout of DHPG, was significantly (*p* < 0.05) inhibited by both LY (80 ± 10%; *n* = 7) and MPEP (82 ± 13%; *n* = 5) compared to the interleaved controls (45 ± 5%; *n* = 7; Figures 2A–D). In contrast, an initial phase of DHPG-LTD^pre^ (short term depression, STD), quantified 5 min after the start of washout of DHPG, was selectively inhibited by LY (82 ± 13%; *p* < 0.05) relative to MPEP (41 ± 8%) or control (38 ± 8%; Figure 2E). DHPG-LTD^post^ was also selectively inhibited by LY (87 ± 9; *n* = 7, *p* < 0.05) compared to MPEP (55 ± 9%; *n* = 6) or control (45 ± 8%; *n* = 8; Figure 2F). A second mGluR1 selective inhibitor, YM298198 (2 μM) similarly inhibited DHPG-LTD^post^ (92 ± 12%; *n* = 7; Figure 2F; Sanderson et al., 2018). Collectively these pharmacological experiments suggest that both mGluR1 and mGluR5 contribute to DHPG-LTD^pre^, which contrasts with the selective involvement of mGluR1 in DHPG-LTD^post^ (Sanderson et al., 2018).

### DHPG-LTD of Miniature Excitatory Postsynaptic Currents

**FIGURE 3:**
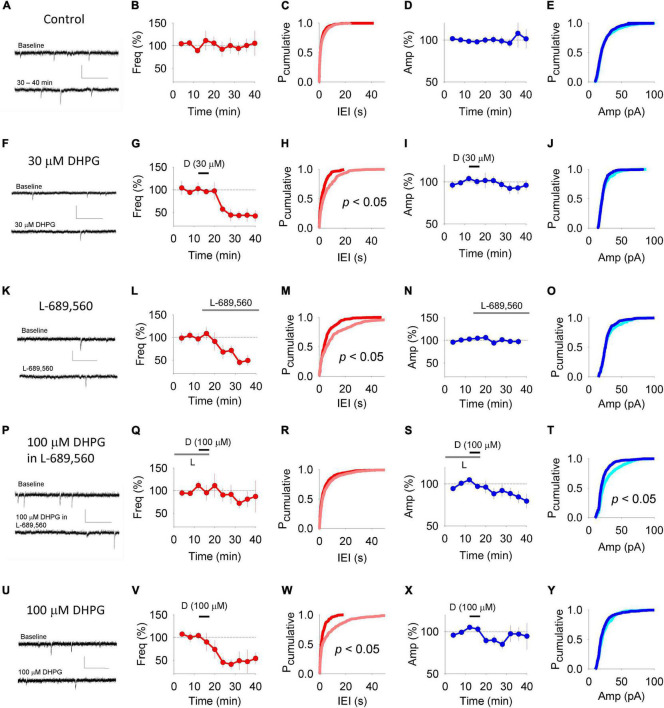
DHPG-LTD of mEPSCs. Example traces of mEPSCs in panel **(A)** control experiments, **(F)** experiments in which 30 μM DHPG was applied alone, **(K)** experiments in which L-689-560 was applied alone, **(P)** experiments in which 100 μM DHPG was applied in 10 μM L-689,560 and **(U)** experiments in which 100 μM DHPG was applied alone. Upper trace taken from baseline period and lower trace taken following treatment. Scale, 0.5 s, 50 pA. For each experiment type, the corresponding averaged mEPSC frequency **(B,G,L,Q,V)** was plotted with respect to time. **(C,H,M,R,W)** Cumulative probability plots showing the inter stimulus intervals between mEPSCs from the baseline (red) and following treatment (pink) from the data sets plotted to the left. Statistical significance determined using Kolmogorov-Smirnov Tests. For each experimental set, the mEPSC amplitude was plotted with respect to time **(D,I,N,S,X)**. **(E,J,O,T,Y)** Cumulative probability plots showing the mEPSC amplitudes from the baseline (light blue) and following treatment (dark blue) from the data sets plotted to the left. Statistical significance determined using Kolmogorov-Smirnov Tests.

Changes to the properties of mEPSCs can also indicate the locus of expression of synaptic plasticity. In control conditions, average mEPSC frequency (100 ± 14% of baseline; *n* = 11; Figures 3A–C) and amplitude (108 ± 17% of baseline; Figures 3A,D,E) were stable over the 40 min time course under study. 30 μM DHPG reduced mEPSC frequency (to 44 ± 14% of baseline at 20 min post the start of washout; Figures 3F,G) and resulted in a rightward shift of cumulative probability distributions of inter-event intervals (*n* = 4; *p* < 0.05, KS test; Figure 3H), without affecting mEPSC amplitude (93 ± 7%; *p* > 0.05; Figures 3I,J).

Consistent with previous reports that NMDARs regulate mEPSC frequency (Berretta and Jones, 1996; Sjostrom et al., 2003; Larsen et al., 2011; Abrahamsson et al., 2017), we found that L-689,560 applied for 20 min depressed mEPSC frequency (to 45 ± 7%; Figures 3K–M) without affecting mEPSC amplitude (Figures 3N,O). We therefore waited until mEPSC frequency had stabilized in L-689,560 before testing the effects of 100 μM DHPG.

The DHPG-LTD^post^ protocol resulted in the opposite profile to that observed for the DHPG-LTD^pre^ protocol. There was no change in mEPSC frequency, both in terms of average values (81 ± 17% of baseline; *n* = 10; Figures 3P,Q) and the cumulative probability distribution of inter-event intervals (*p* > 0.05, KS test; Figure 3R). However, mEPSC amplitude was reduced (to 84 ± 7% of baseline 20 min after DHPG washout; Figure 3S) resulting in a leftward shift in the cumulative probability distribution toward smaller amplitudes (*p* < 0.05, KS test; Figure 3T).

Finally we applied 100 μM RS-DHPG in the absence of L-689,560. This resulted in a reduction in mEPSC frequency (to 47 ± 25% of baseline 20 min post washout; Figures 3U,V) and a corresponding rightward shift of the cumulative probability distribution of inter-event intervals (*n* = 7; *p* < 0.05, KS test; Figure 3W). No change in amplitude was observed according to either measure: Average amplitude was 97 ± 8% of baseline (Figure 3X) and there was no shift in the cumulative probability plot of inter-event intervals (*p* > 0.05, KS test; Figure 3Y).

### Morphological Alterations Accompany Postsynaptically Expressed DHPG-LTD

**FIGURE 4:**
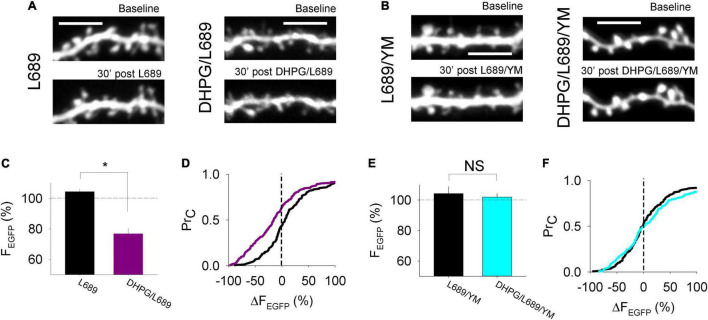
Morphological alterations accompany postsynaptically expressed DHPG-LTD. **(A,B)** Example images of EGFP expressed in CA1 neurons under the conditions shown. **(C)** Average % change in fluorescence integrated density over a 30 min control period where L-689,560 (L689) was applied or 30 min following DHPG washout in ACSF containing L-689,560 (DHPG/L689). Statistical significance determined using an unpaired t-test. *indicates *p* < 0.05. **(D)** Cumulative probability plot of the % change in fluorescence integrated density at individual spines in the L689 condition (black line) and the DHPG/L689 condition (purple line). **(E)** Average % change in fluorescence integrated density over a 30 min control period where L-689,560 and YM298198 were applied (L689/YM) or 30 min following DHPG washout in ACSF containing L689/YM (DHPG/L689/YM). **(F)** Cumulative probability plot of the % change in fluorescence integrated density at individual spines in the L689/YM condition (black line) or in the DHPG/L689/YM condition (cyan line). Statistical significance determined using Kolmogorov-Smirnov tests.

In some (Ramiro-Cortes and Israely, 2013) but not all (Thomazeau et al., 2020) previous studies, DHPG results in morphological alterations to the postsynapse. To determine whether the DHPG-LTD^post^ that we observe is associated with a change in spine size, we transfected organotypic cultures to express EGFP in CA1 neurons and used multiphoton microscopy to image the postsynaptic compartment, while delivering 0.03 Hz stimulation. Compared to interleaved control experiments in which only L-689,560 was applied (*n* = 7) the DHPG-LTD^post^ protocol (*n* = 8) resulted in decreased spine size (integrated pixel density was 104 ± 2% and 77 ± 4% of baseline, respectively; *p* < 0.05; Figures 4A,C) and the cumulative probability distribution was shifted toward smaller sizes (*p* < 0.05, KS test; Figure 4D). This DHPG-LTD^post^ effect was blocked by the mGluR1 antagonist YM298198. Integrated pixel density was 104 ± 5% and 102 ± 2% of baseline in control slices to which only the antagonists had been added (*n* = 5) and DHPG-treated (*n* = 5) neurons, respectively (*p* > 0.05, Figures 4B,E) and the cumulative probability distribution was similar in the 2 conditions (*p* > 0.05, KS test; Figure 4F).

### DHPG-LTD of Evoked Synaptic Transmission in Acute Slices

**FIGURE 5:**
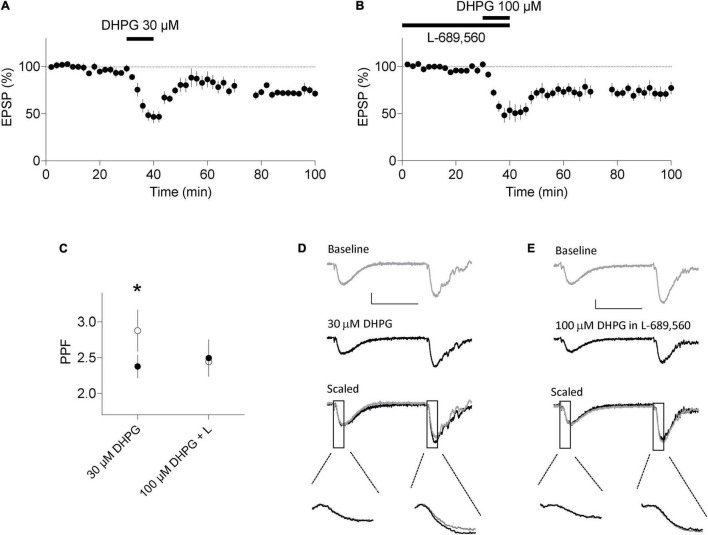
DHPG-LTD of evoked synaptic transmission in acute slices. **(A)** 30 μM RS-DHPG applied for 10 min results in LTD when applied alone. **(B)** 100 μM DHPG applied in the presence of 10 μM L-689,560 results in LTD. **(C)** LTD induced by 30 μM DHPG, but not by 100 μM DHPG in L-689,560 (L) is accompanied by an increase in PPF in response to stimuli with an inter stimulus interval of 50 ms. Filled points, before DHPG. Open points, 60 min following DHPG washout. Statistical significance determined using a paired t-test. *indicates *p* < 0.05. **(D)** Example traces for experiments in A. **(E)** Example traces for experiments in B. Scale bars are 0.2 mV by 25 ms. Lower insets highlight the fEPSP slopes at an expanded time base.

Lastly, we induced LTD in acute slices using the same induction protocols and examined PPF. In interleaved experiments, 60 min after DHPG washout, the DHPG-LTD^pre^ protocol induced LTD to 71 ± 4% of baseline (*n* = 9, *p* < 0.05; Figure 5A) and the DHPG-LTD^post^ protocol resulted in LTD to 77 ± 7% of baseline (*n* = 7; *p* < 0.05; Figure 5B). Consistent with the data from organotypic slices, only the DHPG-LTD^pre^ protocol resulted in an increase in PPF. In these experiments, PPF was 238 ± 15% and 288 ± 29% during baseline and 60 min following DHPG washout, respectively (*p* < 0.05; Figures 5C,D). Whereas in the DHPG-LTD^post^ condition, PPF was 249 ± 26% and 244 ± 18% during baseline and 60 min following washout of DHPG, respectively (*p* > 0.05; Figures 5C,E).

### Model of Synaptic Transmission and PPF

The observation that DHPG-LTD^pre^ and DHPG-LTD^post^ were not additive suggests that they target the same population of synapses and that there exists a second population of synapses that is resistant to the effects of DHPG. Previously we found that the DHPG-LTD^post^ protocol results in AMPAR trafficking specifically at low P(r) synapses, likely due to higher expression of mGluR1 at these synapses (Sanderson et al., 2018). We therefore compared two models of neurotransmitter release in which DHPG reduces P(r) uniformly across all synapses versus only at a subset of low P(r) synapses.

**FIGURE 6:**
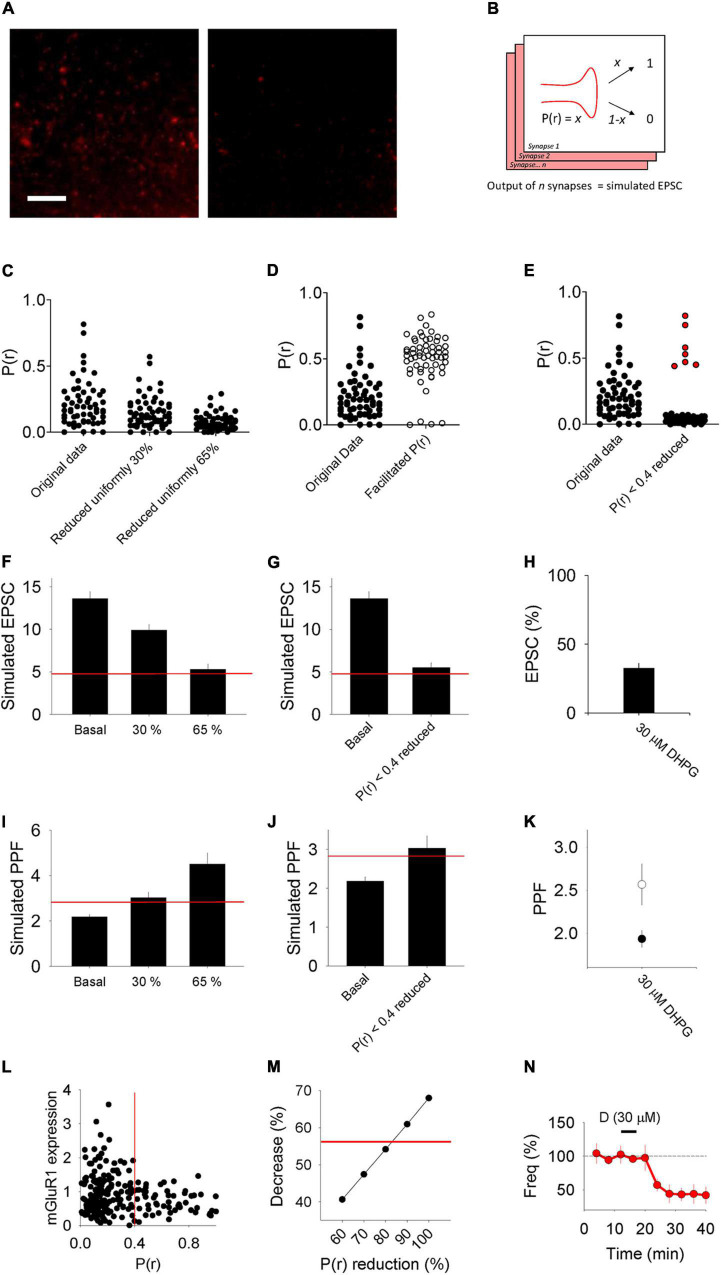
Model of synaptic transmission and PPF. **(A)** Example images of synapses loaded with FM 4–64 to estimate P(r) from Sanderson et al., 2018. Left image: stained. Right image: destained. **(B)** Scheme of model detailing how for each synapse a binary output (e.g., either 0 or 1) is produced according to the P(r) of the synapse. We refer to the sum of the outputs from the 58 synapses used as the “simulated” EPSC. **(C–E)** Plots of P(r) values of 58 individual synapses from Sanderson et al., 2018 (“Original data”) compared, respectively, with plots of manipulations of this data where each point has been reduced by either 30 or 65%, each point has been facilitated according to a model that predicts the P(r) of the second pulse in a PPF experiment (Murthy et al., 1997) and where each point with initial P(r) < 0.4 has been reduced by 80%. Protected synapses [with P(r) > 0.4] plotted in red. **(F,G)** Average simulated EPSCs produced from the original P(r) data (Basal) compared, respectively, with data sets in which the P(r) has been reduced uniformly by 30 and 65% and a data set in which synapses that had initial P(r) < 0.4 have been reduced by 80%. Red horizontal lines represent a 65% reduction from the basal simulated EPSC [the level of LTD seen in *in vitro* DHPG-LTD^pre^ experiments, which is replotted in panel **(H)**]. **(I,J)** Simulated PPF produced from the original P(r) data (Basal) compared with, respectively, data sets in which the P(r) has been reduced uniformly by 30 and 65% and a data set in which synapses that had initial P(r) < 0.4 have been reduced by 80%. Red horizontal lines represent a 30% increase from the basal simulated PPF [the level of PPF seen in *in vitro* DHPG-LTD^pre^ experiments, which is replotted in panel **(K)**]. **(L)** mGluR1 expression levels with respect to P(r). Data replotted from Sanderson et al. (2018). **(M)** Decrease in average P(r) seen when the subset of synapses with initial P(r) < 0.4 have their P(r) reduced incrementally from 60 to 100%. Red line marks a 56% decrease [the decrease seen in DHPG-LTD^pre^ mEPSC recordings, which is replotted in panel **(N)**].

We used a data set of 58 synapses, the P(r) of which was estimated by loading of the styryl dye FM 4-64 in organotypic slices under identical conditions to those used in this study (Figures 6A,C; see Sanderson et al., 2018). We then decreased the P(r) by either 30% at all synapses to reflect the 30% increase in PPF observed in DHPG-LTD^pre^, or by 65% at all synapses to reflect the 65% decrease in synaptic transmission observed in DHPG-LTD^pre^ (Figure 6C). We then produced a model in which for each synapse a binary output (release or no release) was produced according to the P(r) of the synapse, yielding 58 outputs of either 1 or 0 (Figure 6B). The sum of these outputs generated the simulated EPSC. We ran this simulation multiple times to produce the mean and standard error for this data set (Figure 6F). The original data produced a simulated EPSC of 13.6 ± 0.8 (*n* = 10), the 30% reduced condition yielded 9.9 ± 0.7 (*n* = 10) and the 65% reduced condition was 5.3 ± 0.6 (*n* = 10). Only the 65% reduced condition resulted in a similar level of LTD to that observed in the *in situ* experiments (indicated by the red line and replotted in 6H for comparison).

We next sought to model how changes to basal P(r) would influence PPF. To do this we took advantage of a model that relates the initial P(r) of a synapse to the facilitation of P(r) that occurs in PPF (see Methods; Murthy et al., 1997). Using this model we converted the data set of 58 initial P(r) values to the facilitated P(r) that we would expect for the second pulse in a paired-pulse experiment (Figure 6D). We then used these new P(r) values to produce new simulated EPSC data sets that represent the response to the second pulse, and used these to determine a simulated PPF in each condition (Figure 6I). The simulated PPF was 2.2 ± 0.1 in the original condition (*n* = 10), 3.0 ± 0.3 in the 30% reduced condition (*n* = 10) and 4.5 ± 0.5 in the 65% reduced condition (*n* = 10). Only the 30% reduced condition resulted in a similar change in PPF to that observed in the *in situ* experiments (indicated by the red line and replotted in 6K for comparison).

As reducing P(r) uniformly did not produce a combination of changes to both the simulated EPSC and PPF that are consistent with the *in situ* experimental data, we proceeded to test the effect of modulating the P(r) at only a subset of synapses. To determine which subset of synapses to modulate, we turned to our previously published data that indicates that mGluR1 is more highly expressed at synapses with P(r) < 0.4 (Sanderson et al., 2018; replotted in Figure 6L). We therefore chose to modulate synapses with P(r) < 0.4 as these are more likely to be susceptible to this mGluR-dependent form of plasticity. Next, we referred to our mEPSC data (Figure 3) that indicates that DHPG-LTD^pre^ results in a decrease in P(r) of 56% (based on a decrease in mEPSC frequency of this magnitude; Figure 3G. We therefore decreased the P(r) at a subset of synapses with initial P(r) < 0.4, incrementally, from 60 to 100%, and determined the new mean P(r) for each new data set. This approach indicated that decreasing the P(r) of this subset of synapses by ∼80% [and protecting the synapses with higher P(r)] produced an *average* decrease in P(r) most similar to that observed experimentally (Figure 6M, red line). We therefore determined the simulated EPSC and simulated PPF for the condition in which only synapses with P(r) < 0.4 have their P(r) reduced by 80%. We found that in this condition the simulated EPSC was 5.5 ± 0.6 (*n* = 10; Figure 6G) and the simulated PPF was 3.0 ± 0.3 (*n* = 10; Figure 6J). As both values are consistent with the *in situ* experimental data (indicated by the red lines), this modeling is consistent with DHPG-LTD^pre^ as well as DHPG-LTD^post^ (Sanderson et al., 2018), being expressed at a subset of low P(r) synapses.

## Discussion

In the present study we have investigated two distinct forms of LTD induced by the group I mGluR agonist DHPG. One form requires the co-activation of NMDARs and is expressed presynaptically whilst the other is independent of NMDARs and is expressed postsynaptically. Surprisingly, both forms of DHPG-LTD converge on the same set of synapses. We propose that low P(r) synapses, that constitute the majority of synapses at the Schaffer collateral-commissural pathway, are the sensitive population.

The electrophysiological characterization of LTD induced by 30 μM DHPG applied alone is consistent with several previous reports that find that DHPG-LTD is accompanied by changes most easily explained by a presynaptic expression mechanism. These include an increase in PPF, a decrease in mEPSC frequency but not amplitude and no postsynaptic change in response to uncaging of caged glutamate (Fitzjohn et al., 2001a,b; Watabe et al., 2002; Rammes et al., 2003; Rouach and Nicoll, 2003; Tan et al., 2003; Moult et al., 2006; Qian and Noebels, 2006; Sanderson et al., 2011). In contrast LTD induced by 100 μM DHPG applied in the presence of L-689,560 was not accompanied by a change in PPF or a change in mEPSC frequency. However, it was accompanied by a decrease in mEPSC amplitude together with a decrease in spine size. Since mEPSCs are mediated by AMPARs that are expressed at similar densities in spines (Takumi et al., 1999; Tanaka et al., 2005) these results are consistent with the hypothesis that DHPG-LTD^post^ is expressed *via* AMPAR trafficking (Snyder et al., 2001; Xiao et al., 2001; Nosyreva and Huber, 2005; Moult et al., 2006; Sanderson et al., 2011, 2018).

Our findings are in line with reports that the pre and postsynaptic mechanisms can be modulated independently, for example by serotonin receptor agonists (Costa et al., 2012). They are also consistent with our initial report that DHPG can induce LTD *via* two distinct mechanisms, one of which is dependent on NMDARs (Palmer et al., 1997). They do not, however, mean that the NMDAR-dependent and independent forms invariably equate with pre and postsynaptic mechanisms, respectively. For example, a change in PPF has previously been observed in response to DHPG applied in the presence of L-689,560 (Moult et al., 2006). There are a variety of factors that might influence the locus of expression of mGluR-LTD, including a switch from presynaptic to postsynaptic expression mechanisms over the first month of development (Nosyreva and Huber, 2005), a renewed presynaptic contribution in aged animals (Kumar and Foster, 2007) and a greater presynaptic component in slices from the ventral compared to the dorsal hippocampus (Tidball et al., 2017).

We also found that DHPG-LTD^pre^ and DHPG-LTD^post^ have different requirements for the mGluR(s) that trigger the plasticity. We found that DHPG-LTD^pre^ is substantially inhibited by either mGluR1 or mGluR5 antagonists, indicating that both receptors are necessary, possibly in the form of a heterodimer (Werthmann et al., 2021). In contrast, DHPG-LTD^post^ was selectively blocked by mGluR1 antagonists (Sanderson et al., 2018). Previously, roles for mGluR1 or mGluR5, alone or in combination have been reported for mGluR-LTD (Palmer et al., 1997; Fitzjohn et al., 1999; Hou and Klann, 2004; Volk et al., 2006; Moult et al., 2008; Neyman and Manahan-Vaughan, 2008; Nadif Kasri et al., 2011). Our results suggest that different expression mechanisms are related to the types of mGluRs that trigger LTD.

An interesting question is how activation of group I mGluRs generates a form of LTD that requires activation of NMDARs. Since DHPG potentiates the effects of NMDAR activation (Fitzjohn et al., 1996) it is possible that DHPG is permitting sufficient NMDAR activation during basal synaptic stimulation to trigger LTD. An alternative explanation may be that DHPG inhibits a signaling pathway that is maintained by tonically active NMDARs. In this scenario the effects of DHPG and an NMDAR antagonist would occlude one another. Consistent with this hypothesis, application of NMDAR antagonists reduce mEPSC frequency, suggesting that tonically active NMDARs may regulate neurotransmitter release (Berretta and Jones, 1996; Sjostrom et al., 2003; Larsen et al., 2011; Abrahamsson et al., 2017). In our recordings, we also observed that application of L-689,560 caused an initial rundown in mEPSC frequency. Therefore it is possible that the decrease in frequency caused by DHPG and L-689,560 are mutually occlusive.

We hypothesized that applying 100 μM DHPG alone would induce both presynaptic and postsynaptic expression mechanisms, since L-689,560 is not present to block DHPG-LTD^pre^ and 100 μM is a concentration that is able to induce substantial DHPG-LTD^post^. However, when we examined LTD of evoked responses we found no additive effect, e.g., a similar magnitude of LTD was obtained in the presence and absence of L-689,560. In addition we found that 100 μM DHPG alone reduced the frequency without affecting the amplitude of mEPSCs. An explanation for these findings is that the presynaptic and postsynaptic alterations occur at the same subset of synapses, with other synapses protected from the effects of DHPG. Accordingly, DHPG acts to inhibit neurotransmitter release such that postsynaptic changes either do not occur (if they require synaptic activity) or are masked by the presynaptic ones. However, when NMDARs are blocked, the presynaptic effect is eliminated such that a pure postsynaptic form of mGluR-LTD remains.

Previously we investigated the postsynaptic expression mechanisms involved in DHPG-LTD^post^ and found that in this form of plasticity high P(r) synapses are resistant to weakening *via* AMPAR trafficking because the receptor that triggers it, mGluR1, is down regulated at high P(r) synapses (Sanderson et al., 2018). In order to investigate if high P(r) synapses are also resistant to DHPG-LTD^pre^ we performed modeling to study how reducing the P(r) of synapses would be expected to change synaptic transmission and PPF. We found that the characteristics of our *in vitro* DHPG-LTD^pre^ experiments (a 65% decrease in synaptic transmission and a 30% increase in PPF), were not reproduced by decreasing the P(r) of all synapses uniformly, however, they were reproduced if we decreased the P(r) of a subset of low P(r) (<0.4) synapses (Figure 6). This modeling is therefore consistent with the hypothesis that high P(r) synapses are resistant to the presynaptic expression mechanisms as well as the postsynaptic mechanisms.

The size and functionality of the pre and postsynapse are correlated (Sanderson et al., 2020) in part through activity-dependent regulation of group I mGluR receptors (Sanderson et al., 2018). If both the presynaptic and postsynaptic mechanisms occur at the same subset of low P(r) synapses it is possible that they could play a role in maintaining pre and postsynaptic complementarity when induced together. During sleep, synapses are downregulated in a process that spares the strongest (de Vivo et al., 2017) and that requires group I mGluR-induced AMPAR trafficking (Diering et al., 2017). Therefore sleep may be the context in which the mechanisms we have studied here are engaged.

In summary, we have demonstrated how two forms of mGluR-LTD with distinct expression mechanisms can be induced by the application of DHPG. The existence of multiple mechanisms may explain some of the controversies surrounding the roles of various signaling molecules and the requirement for *de novo* protein synthesis in mGluR-LTD (Osterweil et al., 2010; Sharma et al., 2010). Such details are important to note since DHPG is still widely used to study synaptic plasticity in a variety of disease models.

## Data Availability Statement

The raw data supporting the conclusions of this article will be made available by the authors, without undue reservation.

## Ethics Statement

The animal study was reviewed and approved by the Animal Care and Use Committee of Seoul National University and UK Animal Scientific Procedure Act 1986.

## Author Contributions

TS performed and analyzed the majority of the experiments in organotypic slices, performed the modeling, wrote the first draft of the manuscript, and edited the manuscript. LR performed and analyzed the experiments in acute slices. MA performed and analyzed some of the experiments in organotypic slices. AN analyzed the imaging experiments. B-KK, MZ, and SK co-supervised the project. JG edited the manuscript. GC conceived and supervised the study and edited the manuscript. All authors contributed to the article and approved the submitted version.

## Conflict of Interest

The authors declare that the research was conducted in the absence of any commercial or financial relationships that could be construed as a potential conflict of interest.

## Publisher’s Note

All claims expressed in this article are solely those of the authors and do not necessarily represent those of their affiliated organizations, or those of the publisher, the editors and the reviewers. Any product that may be evaluated in this article, or claim that may be made by its manufacturer, is not guaranteed or endorsed by the publisher.
